# Predictors of Energy Compensation during Exercise Interventions: A Systematic Review

**DOI:** 10.3390/nu7053677

**Published:** 2015-05-15

**Authors:** Marie-Ève Riou, Simon Jomphe-Tremblay, Gilles Lamothe, Dawn Stacey, Agnieszka Szczotka, Éric Doucet

**Affiliations:** 1 Behavioural and Metabolic Research Unit (BMRU), School of Human Kinetics, Faculty of Health Sciences, University of Ottawa, Ottawa, Ontario K1N 6N5, Canada; E-Mails: mriou039@uottawa.ca (M.-E.R.); sjomp103@gmail.com (S.J.-T.); 2 Department of Mathematics and Statistics, Faculty of Science, University of Ottawa, Ontario K1N 6N5, Canada; E-Mail: glamothe@uottawa.ca; 3 Ottawa Hospital Research Institute, University of Ottawa, Ontario K1Y 4M9, Canada; E-Mail: Dawn.Stacey@uOttawa.ca; 4 School of Nursing, Faculty of Health Sciences, University of Ottawa, Ontario, K1H 8 M5, Canada; 5 Health Sciences Library, University of Ottawa, Ottawa, Ontario K1H 8M5, Canada; E-Mail: agnieszka.szczotka@uottawa.ca

**Keywords:** energy compensation, body composition, exercise intervention

## Abstract

Weight loss from exercise-induced energy deficits is usually less than expected. The objective of this systematic review was to investigate predictors of energy compensation, which is defined as body energy changes (fat mass and fat-free mass) over the total amount of exercise energy expenditure. A search was conducted in multiple databases without date limits. Of 4745 studies found, 61 were included in this systematic review with a total of 928 subjects. The overall mean energy compensation was 18% ± 93%. The analyses indicated that 48% of the variance of energy compensation is explained by the interaction between initial fat mass, age and duration of exercise interventions. Sex, frequency, intensity and dose of exercise energy expenditure were not significant predictors of energy compensation. The fitted model suggested that for a shorter study duration, lower energy compensation was observed in younger individuals with higher initial fat mass (FM). In contrast, higher energy compensation was noted for younger individuals with lower initial FM. From 25 weeks onward, energy compensation was no longer different for these predictors. For studies of longer duration (about 80 weeks), the energy compensation approached 84%. Lower energy compensation occurs with short-term exercise, and a much higher level of energy compensation accompanies long-term exercise interventions.

## 1. Introduction

Obesity results from a long-term mismatch between readily-available energy-dense and palatable food and low levels of daily energy expenditure (EE) that characterizes our modern way of life [1]. In order to promote weight loss, diets over a short period of time lead to successful results, although weight regain is noted in 97% of the cases after dietary-induced weight losses [2]. For exercise-induced weight loss, the results are often much less than anticipated. Indeed, in a meta-analysis done in the late 1990s, it was reported that the impact of exercise on body weight changes is usually less than 2–3 kg of the initial body weight [3], a weight loss similar to that noted in more recent reviews and/or meta-analyses [4,5]. Since the observed weight loss is often much less than what could be anticipated from the dose of exercise, this implies that some form of energy compensation, *i.e.*, increased energy intake (EI), decreased energy expenditure, a small dose of exercise induced energy expenditure (ExEE) [6] or simply a lack of compliance to the prescribed exercise [7], is occurring.

To examine the impact of exercise on body energy stores, body weight has often been the main target [8]. However, this variable does not take into account the individual and independent variation of fat-free mass (FFM) and fat mass (FM) [9]. Therefore, body composition rather than body weight changes have to be investigated as a function of the ExEE in order to allow a fair comparison between studies [9]. Accordingly, a relative measure (energy compensation) of the response to exercise that accounts for body composition changes as a function of ExEE has been used in a very limited number of studies [9]. However, the contributions of sex [10,11,12,13] and adiposity to energy compensation [14,15,16,17] remain contradictory and deserve more attention. Regarding the impact of age, the elderly have been shown to decrease their non-structured physical activity following exercise [18,19,20]. However, as pointed out by Melanson and colleagues [21], none of these studies have compared the impact of exercise on energy compensation in a study design comparing younger and older individuals. Similarly, the effects of dose (kcal/week) [9,22,23] and intensity of exercise [24,25] have not been clearly established as far as energy compensation in response to exercise is concerned. Finally, the frequency (session/week) and duration of exercise interventions (week) [26] also need to be investigated to allow a better understanding of ExEE on energy compensation. 

The purpose of this systematic review was to determine the energy compensation following exercise interventions. The contributions of sex, age, initial adiposity, as well as duration, dose, frequency and intensity of exercise on energy compensation remain largely unknown. Therefore, for the first time, the independent contributions of these predictors, as well as their interactions were investigated. It was hypothesized that exercise interventions would lead to positive energy compensation and that sex, intensity and the duration of the exercise would be the strongest predictors of energy compensation. More specifically, we proposed that women would show greater energy compensation when compared to men and that a longer duration of intervention and higher intensity would lead to higher energy compensation.

## 2. Experimental Section

### 2.1. Search Protocol

A literature search was completed in August 2013. The search strategy included a combination of key words and controlled vocabulary related to body weight and body composition changes across the exercise interventions (*i.e.*, fat mass (FM) and (FFM)), maximal aerobic capacity (VO_2peak_), ExEE and aerobic exercise). A librarian performed a literature search in the following databases: MEDLINE (Ovid MEDLINE(R) In-Process & Other Non-Indexed Citations and Ovid MEDLINE(R) 1946 to 2013 (Ovid (Appendix 1)), Embase (Embase Classic and Embase 1947 to August 2013 (Ovid)), Cochrane Central Register of Controlled Trials September 2013 (Ovid), Cinahl (Ebsco), SPORTDiscus (Ebsco) and Physical Education Index (Proquest). Filters listed in the exclusion criteria table were added to limit and specify the search. A detailed list of all inclusion and exclusion criteria for the search is presented in Table 1.

### 2.2. Article Selection Process

From the search protocol, 4745 articles corresponding to the specific key words and controlled vocabulary were found. In order to ensure that each of these articles met the different inclusion and exclusion criteria, a selection process was performed on a web portal by two authors (MER and SJT). The selection process was sequentially applied to all article titles, followed by the abstracts of the articles for which the title was not excluded and then to the full articles for which the abstract and the title were not excluded. Every title, abstract and article was revised independently by each author. To exclude a title/abstract/article from the set, both reviewers had to agree that it met one of the exclusion criteria (*i.e.*, elderly, acute exercise only). Similarly, both authors had to agree that it did not meet any exclusion criteria in order to keep the article for the following phase. When the authors disagreed, the titles/abstract/article was categorized as “unsure” and kept for the following phase. At the last phase (articles screening phase), when ambiguities in the article remained (*i.e.*, impossibility to obtain ExEE, possibility of the use of a dietary intervention), it was discussed and validated with a third party (ÉD). Finally, a database with the full articles was created using an Excel spreadsheet. The full articles were printed, and the two authors separately reviewed all of them. When both authors rejected an article, the main reason was written on the article. It was classified according to reason for exclusion in order to keep a record of the excluded articles. The reasons for rejecting articles were documented. Additional articles found from reviews and/or articles in the bibliography were also added and fully revised (*n* = 13). Throughout the screening process, duplicates were removed (*n* = 43).

**Table 1:** Criteria of included and excluded items for study selection. FFM, fat-free mass; FM, fat mass; EE, energy expenditure; NSPA, non-structured physical activity.

Criteria	Included	Excluded
Population	Men and Women Aged from 18–55 years old Any BMI Women with a period on a regular basis Healthy individual	Under 18 or over 55 years old Menopausal women Illness (type 2 diabetes, hypertension, cancer, hyperinsulinemia) Athletes or military Smoker, drinker (>2 drinks/day) or individual with drug abuse Under medication
Focus/intervention	Aerobic training Interval training Any intervention time intervention duration	Yoga Stretching program Resistance training/callisthenic exercise Animal intervention Diet, caloric restriction and dietary or vitamin supplement Nutrition or cognitive counselling Intervention that aim to maintain or increasing NSPA
Outcomes	Body weight	Maximal heart rate
	FFM	
	FM	
	EE	
	VO_2max_ reserve	
	Maximal heart rate reserve	
Study design	RCTs Pre- and post-test design Interrupted time series	
Language	English French	Other languages
Publication status	Published articles (including all years)	Unpublished articles Undergoing publication process Abstract only available

### 2.3. Synthesis Process: Body Composition, EE Related to Exercise and Degree of Energy Compensation (%)

Data on body composition (FM and FFM) changes were calculated by subtracting the pre-exercise from the post-intervention values. ExEE was obtained directly from the text of the articles (ExEE per session or for the overall study) or through the following calculations, when all data were available:

(1) $$Estimated\;EE\;\left(kcal\right)=VO_{2peak}\left(\frac{L}{kg*min}\right)*weight\;\left(kg\right)*time\;\left(min\right)*5\frac{kcal}{L}$$

Articles lacking ExEE or the data needed to calculate ExEE, as described above, were excluded from this review (*i.e.*, no ExEE, no precise measure of EE or the mention of % heart rate (HR)_max_ only) (Table 1).

The degree of energy compensation was calculated from the ExEE (kcal) and body composition changes (kg converted to kcal) over the course of the exercise intervention. Changes were calculated by subtracting body composition values obtained at the end of the intervention from those measured at baseline. As such, a negative value is indicative of reductions in energy stores. The changes in body energy were calculated using the equivalents described by Hall (2008) [27], where a gain/loss of 1 kg of FM corresponds to 9500 kcal, while it corresponds to 1200 kcal for FFM. The degree of energy compensation (%) was calculated using the following equation:

(2) $$\text{Degree of energy compensation = }\frac{100}{Energy\;Expenditure\;from\;Exercise\;\left(kcal\right)}*\left[\left(\Delta FM\left(kg\right)*9,500\;(\frac{kcal}{kg})\right)+(\left(\Delta FFM\left(kg\right)*1,200\;(\frac{kcal}{kg})\right)\right]+100$$

A compensation of 0% is indicative of the fact that body composition varied perfectly as a function of ExEE. In contrast, a compensation of 100% indicates that body composition remained the same despite ExEE. Finally, when compensation is negative, then body energy stores are reduced beyond what is expected from the amount of energy spent during exercise.

### 2.4. Statistical Analysis

Findings are presented as the mean ± SD. Statistical analyses were performed using SPSS software (Version 21; SPSS Inc., Chicago, IL, USA) and with R (Version 3.0.1). Results were considered significant at *p* < 0.05. The studies included were weighted for the number of participants in each study. Linear models were used to compare the degree of energy compensation between groups (sex and intensity) and to determine the association between the degree of energy compensation and the following predictors: initial FM, age, dose of exercise, duration of the intervention and frequency. 

A general linear model with interactions was constructed to determine the significant predictors of the degree of energy compensation (%). Factors with fixed effects were sex, initial FM (kg), initial BMI (kg/m^2^), age (y), intensity (low *vs*. high), frequency (sessions/week), dose (kcal/week) and duration of exercise intervention (week). Initial FM, BMI, frequency, age and dose of exercise, as well as the duration of exercise intervention were entered into the model as continuous factors. Sex and intensity (two groups divided on the basis of exercise intensity lower or equal to/higher than 60% of VO_2max_, HRmax or HRreserve [28]) were entered into the model as categorical factors. The variable intensity was divided into high and low, because not all of the studies provided accurate values of measured cardiorespiratory assessments. Random effects were attributable to the different studies. 

Before the construction of the model, studies that included men and women, but that did not provide independent results for each of the sexes were not included. Furthermore, studies that did not provide body composition measured with either dual-X-ray absorptiometry (DEXA), hydrostatic weighing or bod pod were excluded. Only articles with mean age or with a small range of age (*i.e.*, [19,20,21,22,23]) were kept for further investigation. One group was discarded because the frequency was not mentioned in the article (total articles included = 40). Based on the degree of energy compensation formula, we used the inverse of the frequency, dose and duration of exercise intervention to better fit the model. To assess variance inflation due to the multicollinearity of the predictors, we used linear regressions to examine the association between the continuous predictors. Since initial FM and BMI were strongly associated (*R*-squared = 0.89; *p* < 0.0001), initial BMI was not further used in the model. This decision was mostly based on the fact that several missing data were noted for this variable and because FM is a more accurate measure of adiposity. Since the inclusion of second order terms, such as interaction terms and quadratic terms, in the model can cause variance inflation due to multicollinearity, continuous predictors to include second order terms were standardized [29]. 

The model was initially fit with a weighted least squares using the number of participants in the study as the weight. The fit of the model was visually assessed with a Q-Q plot and a residual plot of the weighted residuals. In the model with no interactions, only inverse length (*p* < 0.0001) and initial FM (*p* < 0.05) were significant. There was a trend for age. As we included the interactions for these predictors in the model, the interactions were significant. Intervention duration (*F*(3,50) = 14.66; *p* < 0.0001), age (*F*(3,50) = 6.65; *p* < 0.0007) and initial FM (*F*(3,50) = 8.73; *p* < 0.0001) were significant. Neither sex (*F*(1,50) = 0.42; *p* = 0.52), nor frequency (*F*(1,50) = 0.10; *p* = 0.76), nor dose (*F*(1,50) = 0.214; *p* = 0.64), nor intensity *F*(1,50) = 0.43; *p* = 0.51) were significant; thus, they were dropped from the model. The reduced interaction model was fitted with a weighted least squares and was highly significant (*F*(6,54) = 10.18; *p* < 0.0001). In order to determine significant differences, *post hoc* test analyses were performed using general linear tests.

## 3. Results

**Figure 1:**
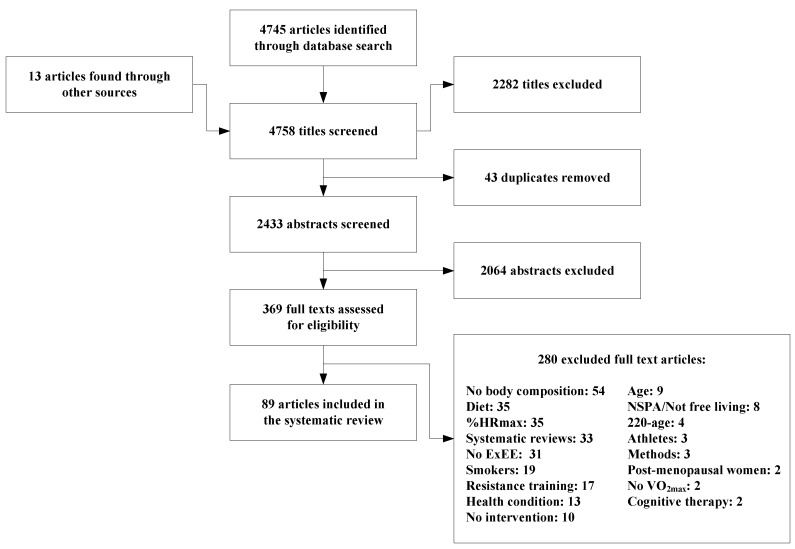
Flow diagram of the screening process. From the 89 studies selected, 71 were from original studies and 18 were from secondary data analyses of the 71 papers that were included. From the 71 studies, 61 groups were used in the final analysis. For these 61 groups, results were presented for each sex (*n* = 26 male; *n* = 35 female), and body composition was measured with either dual-X-ray absorptiometry (DEXA), hydrostatic weighing or bod pod. Only articles with a mean age or with a small range of age (*i.e.*, [19,20,21,22,23]) were kept for further investigation. One group was discarded because the frequency was not mentioned in the article.

The overall characteristics of the studies included in this review and the baseline characteristics of the participants are presented in Table 2. Table 3 presents the characteristics of the interventions and the outcomes of the different studies. The risks of bias are also illustrated in Appendix 2. For most of the studies, the risk of bias was either characterised as a lower or unclear risk. Results suggest that higher risk was found for random sequence generation (~64%). As for other biases, more than 75% of the studies were classified as moderate or high risk. The reasons were minor and mostly related to a lack of information regarding energy intake and non-structured physical activity that could have helped to explain the degree of energy compensation. Other reasons also included the compliance of the participants. This systematic review included a total of 89 studies (Figure 1). After close inspection, 18 studies from the 89 studies were excluded, because they consisted of secondary data analysis of studies already included in this systematic review. Then, after, these 71 studies were subdivided into 101 groups (*i.e*., re-divided on the basis of sex, intensity), which included a total of 1565 subjects. From the 71 studies, 61 groups were used in the final analysis. For these 61 groups, results were presented for each sex (*n* = 26 male; *n* = 35 female), and body composition was measured with either dual-X-ray absorptiometry (DEXA), hydrostatic weighing or bod pod. Only articles with mean age or with a small age range (*i.e.*, [19,20,21,22,23]) were kept for further investigation. One group was removed because the frequency was not mentioned in the article.

Analyses revealed no significant difference in the degree of energy compensation between men and women (21.4% ± 61.2% and 16.1% ± 109.1%, respectively (*p* = 0.83)). When considering the intensity of the interventions, there was no significant difference for the degree of energy compensation between lower (11.8% ± 122.6%) and higher intensity (20.4% ± 81.4%) (*p* = 0.75) (*n* = 61 groups). To further investigate the relationship between continuous variables and the degree of energy compensation, linear regressions were performed. A significant positive correlation between the degree of energy compensation and the duration of the exercise interventions was observed, suggesting that exercise performed over a longer period leads to a higher degree of energy compensation (*r* = 0.30, *p* < 0.002) (*n* = 61 groups). Age (*p* = 0.12) (*n* = 61 groups), frequency (*p* = 0.23) (*n* = 61 groups), initial FM (*p* = 0.12) (*n* = 61 groups) and dose of exercise (*p* = 0.88) (*n* = 61 groups) were not correlated with the degree of energy compensation.

**Table 2:** Characteristics of included studies and baseline participants (*n* = 61).

Studies		Studies Characteristics	*n*	Participants Characteristics at Baseline					
First Author	Year	Group Design	Inclusion	Sex	Sedentary	Stable Body Weight	VO_2peak_ (mL/kg/min)	Age (year)	Weight (kg)
Abe [30]	1997	RCT	9	female	Yes	N/A	N/A	19–23	54.5 ± 4.9 (9)
Blaney [31]	1991	Before-after	7	male	Yes	N/A	32.4 ± N/A	42.0 ± 6.0	91.0 ± 15.0 (7)
Brandon [32]	2006	RCT	28	female	Yes	Yes	32.0 ± N/A	37.3 ± N/A	85.6 ± N/A (28)
Carter [33]	2001	Before-after	8	male	N/A	N/A	41.5 ± 6.7	22.0 ± 1.0	78.1 ± 7.2 (8)
Carter [33]	2001	Before-after	8	female	N/A	N/A	31.9 ± 3.9	22.0 ± 2.0	68.2 ± 7.0 (8)
Caudwell [12] ^1^	2013	ITS	35	male	Yes	Yes	34.9 ± 6.9	41.3 ± 8.6	96.9 ± 13.2 (35)
Caudwell [12] ^1^	2013	ITS	72	female	Yes	Yes	29.1 ± 6.5	40.6 ± 9.5	85.9 ± 11.5 (72)
Cowan [34]	1985	RCT	16	female	Yes	N/A	N/A	41.3 ± 4.4	67.5 ± 11.2 (16)
Cramer [35]	1991	RCT	25	female	N/A	N/A	25.7 ± 0.9	36.0 ± 1.6	76.5 ± 1.9 (18)
Després [36]	1991	Before-after	13	female	N/A	N/A	24.3 ± N/A	38.8 ± 5.3	90.0 ± 11.8 (13)
Donnelly [37]	2000	ITS	11	female	Yes	N/A	23.6 ± 2.8	54.0 ± 9.0	81.4 ± 5.7 (11)
Donnelly [37]	2000	ITS	11	female	Yes	N/A	22.9 ± 4.1	49.0 ± 8.0	85.9 ± 13.1 (11)
Donnelly [38]	2013	RCT	32	female	N/A	N/A	31.6 ± 3.8	22.6 ± 3.2	81.3 ± 13 (18)
Donnelly [38]	2013	RCT	31	female	N/A	N/A	29.8 ± 4.1	22.6 ± 2.9	83.3 ± 18.9 (19)
Donnelly [38]	2013	RCT	30	male	N/A	N/A	36.4 ± 6.4	23.3 ± 3.7	102. 0 ± 11.7 (19)
Donnelly [38]	2013	RCT	22	male	N/A	N/A	37.1 ± 6.5	23.5 ± 3.2	99.9 ± 19.4 (18)
Dowdy [39]	1985	Before-after	18	female	Yes	N/A	33.8 ± 3.9	31.5 ± 5.6	63.4 ± 7.2 (18)
Earnest [40] ^2^	2013	Before-after	21	male	Yes	N/A	29.5 ± 2.9	48.0 ± 9.0	93.9 ± 9.6 (21)
Earnest [40]	2013	Before-after	21	male	Yes	N/A	28.3 ± 4.5	49.0 ± 9.0	98.9 ± 12.7 (16)
Glisezinski [41]	2003	Before-after	11	male	N/A	Yes	34.3 ± 1.3	25.6 ± 1.4	89.5 ± 1.6 (11)
Glowacki [42]	2004	RCT + ITS	N/A	male	Yes	N/A	40.8 ± 9.0	25.0 ± 5.0	87.9 ± 16.6 (12)
Grediagin [24]	1995	Before-after	9	female	Yes	Yes	31.5 ± 3.8	30.0 ± 5.0	68.2 ± 5.9 (6)
Grediagin [24]	1995	Before-after	9	female	Yes	Yes	31.3 ± 3.3	31.0 ± 6.0	68.6 ± 4.6 (6)
Hardman [43]	1992	ITS	34	female	Yes	N/A	N/A	44.9 ± 1.5	64.0 ± 1.7 (28)
Hinkleman [44]	1993	RCT	25	female	N/A	N/A	25.7 ± 0.9	36.0 ± 1.6	76.5 ± N/A (18)
Juneau [45]	1987	RCT	30	male	Yes	N/A	31.9 ± 4.4	49.0 ± 6.0	79.4 ± 11.0 (28)
Juneau [45]	1987	RCT	30	female	Yes	N/A	25.8 ± 3.9	47.0 ± 5.0	63.8 ± 8.0 (24)
Kirk [46]	2003	RCT	N/A	female	Yes	N/A	32.8 ± 4.2	24.0 ± 5.0	77.0 ± 11.4 (25)
Kirk [46]	2003	RCT	N/A	male	Yes	N/A	39.2 ± 5.2	22.0 ± 4.0	94.0 ± 12.6 (16)
Krustrup [47]	2010	RCT	25	female	Yes	N/A	32.7 ± 1.1	37.0 ± 2.0	71.6 ± 2.3 (21)
Krustrup [47]	2010	RCT	25	female	Yes	N/A	35.5 ± 1.4	37.0 ± 1.0	67.1 ± 1.8 (17)
Krustrup [48]	2009	RCT	13	male	Yes	N/A	39.6 ± 1.5	30.0 ± 2.0	82.2 ± 2.9 (12)
Krustrup [48]	2009	RCT	12	male	Yes	N/A	39.3 ± 2.5	31.0 ± 2.0	85.8 ± 5.5 (10)
Lee [49]	2009	RCT	10	male	N/A	Yes	46.2 ± 1.2	26.2 ± 1.4	73.8 ± 2.1 (9)
Moro [50]	2005	Before-after	10	male	N/A	Yes	34.7 ± 1.2	26.0 ± 1.4	90.3 ± 1.6 (10)
Mougios [25]	2006	Before-after	7	female	Yes	Yes	36.6 ± 3.8	30.0 ± 9.0	64.1 ± 6.3 (7)
Mougios [25]	2006	Before-after	7	female	Yes	Yes	34.0 ± 5.6	31.0 ± 9.0	68.7 ± 8.7 (7)
Nishida [51]	2010	Before-after	6	male	Yes	N/A	41.3 ± 2.0	24.5 ± 1.9	66.4 ± 3.5 (6)
Nordby [52] ^3^	2012	RCT	17	male	Yes	Yes	38.2 ± 1.7	28.0 ± 1.0	94.5 ± 2.3 (12)
Nybo [53]	2010	Before-after	9	male	Yes	N/A	39.3 ± 2.5	31.0 ± 2.0	85.8 ± 5.5 (9)
Polak [54]	2006	Before-after	25	female	Yes	Yes	24.6 ± 3.9	40.4 ± 6.7	88.5 ± 8.2 (25)
Rosenkilde [9] ^4^	2012	RCT	21	male	Yes	Yes	34.6 ± 4.1	30.0 ± 7.0	93.2 ± 8.1 (18)
Rosenkilde [9] ^4^	2012	RCT	22	male	Yes	Yes	36.2 ± 5.3	28.0 ± 5.0	91.3 ± 7.2 (18)
Ruby [55]	1996	Before-after	6	female	Yes	N/A	39.9 ± 1.2 ^8^	20.3 ± 0.9	58.2 ± 3.3 (6)
Ruby [55]	1996	Before-after	6	female	Yes	N/A	33.6 ± 0.2 ^8^	20.5 ± 1.0	61.6 ± 3.6 (6)
Ruby [55]	1996	Before-after	6	female	Yes	N/A	36.8 ± 1.4 ^8^	21.3 ± 0.6	62.4 ± 3.0 (6)
Santiago [56]	1995	RCT	21	female	Yes	N/A	31.5 ± 4.2	30.1 ± 5.3	64.4 ± 10.2 (16)
Sedlock [57]	2010	RCT	10	male	N/A	Yes	46.2 ± 1.2	26.2 ± 1.4	73.8 ± 2.1 (9)
Sijie [58]	2012	RCT	20	female	N/A	N/A	33.3 ± 3.9	19.8 ± 1.0	73.7 ± 7.5 (17)
Sijie [58]	2012	RCT	20	female	N/A	N/A	32.9 ± 4.7	19.3 ± 0.7	74.2 ± 9.0 (16)
Snyder [59]	1997	Before-after	15	female	Yes	Yes	24.0 ± 4.6	43.0 ± 11.0	87.2 ± 21.5 (13)
Suter [60]	1995	Before-after	20	male	Yes	N/A	39.3 ± 5.5	39.1 ± 8.3	75.6 ± 9.8 (12)
Tan [61]	2012	RCT	30	female	Yes	N/A	34.1 ± 2.6	20–23	70.4 ± 5.3 (29)
Trapp [62]	2008	RCT	15	female	Yes	N/A	28.8 ± 2.1	22.4 ± 0.7	63.3 ± 3.8 (11)
Trapp [62]	2008	RCT	15	female	Yes	N/A	30.9 ± 2.1	21.0 ± 0.8	59.8 ± 2.4 (8)
Van Aggel-Leijssen [63]	2002	RCT	8	male	Yes	Yes	31.1 ± N/A	43.4 ± 6.3	102.7 ± 10.8 (8)
Van Aggel-Leijssen [63]	2002	RCT	8	male	Yes	Yes	31.4 ± N/A	40.0 ± 6.3	105.5 ± 6.6 (8)
Van Aggel-Leijssen [64]	2001	Before-after	8	female	Yes	Yes	24.7 ± N/A	32.8 ± 9.6	91.2 ± 9.7 (8)
Van Aggel-Leijssen [64]	2001	RCT	7	female	Yes	Yes	24.6 ± N/A	37.7 ± 6.4	86.5 ± 10.2 (7)
Wilmore [65]	1980	RCT	9	male	Yes	N/A	38.6 ± N/A	37.0 ± 8.9	85.7 ± 18.9 (9)
Wilmore [65]	1980	RCT	9	male	Yes	N/A	42.2 ± N/A	35.6 ± 8.3	79.8 ± 8.9 (9)

The values are the mean ± SD or presented as a range. The number in parentheses represents the number of participants tested. Notes. RCT, randomised controlled trial; ITS, interrupted time series; ^1^ trial registration: ISRCTN47291569; ^2^ trial registration: PBRC29018; ^3^ trial registration: NCT01090869; ^4^ trial registration: NCT01430143.

**Table 3:** Characteristics of the intervention and the outcomes (*n* = 61). ExEE, exercise EE.

Studies	Interventions Characteristics			Outcomes of the Interventions						
First Author, year	Supervised	Compliance (%)	Exercise Intervention	Measure of BC	FM (Initial) (kg)	FM (Final) (kg)	FFM (Initial) (kg)	FFM (Final) (kg)	ExEE Total (kcal)	Compensation (%)
Abe, 1997 [30]	Yes	N/A	2.8×/week for 30 min during 13 weeks of continuous biking at 50%–60% HRR_max_	HW	15.3 ± 2.7 (9)	12.7 ± 2.1 (9)	39.2 ± 3.3 (9)	38.5 ± 3.4 (9)	7280	−245
Blaney, 1991 [31]	No	N/A	3×/week for 28 min during 16 weeks of continuous running/walking at 70%–80% VO_2max_	HW	25.5 ± N/A (7)	23.7 ± N/A (7)	65.0 ± 10.0 (7)	67.0 ± 10.0 (7)	16,131	14
Brandon, 2006 [32]	No	87.6	3×/week for 50 min during 18 weeks of continuous brisk walking at a self-pace with an objective of 3.5 mph	DEXA	38.5 ± N/A (28)	36.1 ± N/A (28)	47.1 ± N/A (28)	47.6 ± N/A (28)	17,521	−24
Carter, 2001 [33]	No	N/A	5×/week for 60 min during 7 weeks of continuous biking at 60% VO_2peak_	DEXA	12.2 ± N/A (8)	11.8 ± N/A (8)	65.9 ± 7.1 (8)	66.0 ± 6.6 (8)	22,191	84
Carter, 2001 [33]	No	N/A	5×/week for 60 min during 7 weeks of continuous biking at 60% VO_2peak_	DEXA	17.9 ± N/A (8)	17.2 ± N/A (8)	50.3± 4.2 (8)	50.3 ± 4.1 (8)	15,769	58
Caudwell, 2013 [12]	Yes	N/A	5×/week during 12 weeks of continuous walking/biking/running/rowing/stepping machine at 70% HR_max_	BP	33.2 ± 10.4 (35)	30.1 ± N/A (35)	63.4 ± 6.5 (35)	63.5 ± N/A (35)	29,339	0
Caudwell, 2013 [12]	Yes	N/A	5×/week during 12 weeks of continuous walking/biking/running/rowing/stepping machine at 70% HR_max_	BP	38.3 ± 9.0 (72)	35.3 ± N/A (72)	47.7 ± 5.8 (72)	48.3 ± N/A (72)	27,547	1
Cowan, 1985 [34]	No	93.75	4×/week for 17–44 min during 9 weeks of continuous walking at 80% aged predicted HR_max_	HW	21.9 ± N/A (16)	21.2 ± N/A (16)	45.6 ± N/A (16)	46.3 ± N/A (16)	6001	11
Cramer, 1991 [35]	Yes	100	5×/week for 45 min during 15 weeks of continuous walking/biking at 62% VO_2max_	HW	27.9 ± N/A (18)	27.8 ± N/A (18)	48.6 ± N/A (18)	48.7 ± N/A (18)	20,810	94
Després, 1991 [36]	No	N/A	4.5×/week for 90 min during 61 weeks of continuous walking/biking/aerobic dance/swimming at 55% VO_2max_	HW	42.6 ± 9.4 (13)	38.0 ± 7.3 (13)	47.4 ± 5.1 (13)	48.3 ± 4.1 (13)	163,327	74
Donnelly, 2000 [37]	Yes	91.9	3×/week for 29 min during 78 weeks of continuous exercise (N/A) at 60%–75% VO_2max_	HW	34.0 ± 3.7 (11)	31.9 ± 3.3 (11)	47.4 ± 3.7 (11)	47.8 ± 3.8 (11)	41,793	54
Donnelly, 2000 [37]	Not always	90.3	5×/week for 14.5 min twice daily during 78 weeks of continuous walking at 50%–65% HR_reserve_	HW	36.7 ± 7.0 (11)	36.0 ± 7.7 (11)	49.1 ± 7.7 (11)	49.1 ± 7.5 (11)	60,492	89
Donnelly, 2013 [38]	Yes	>90	5×/week for the time necessary to expend 600 kcal/session during 43.5 weeks of continuous biking/running/walking/exercise on elliptical machine at 70%–80% HR_max_	DEXA	34.1 ± 9.4 (18)	29.7 ± 9.6 (18)	46.1 ± 5.3 (18)	46.9 ± 4.8 (18)	111,703	64
Donnelly, 2013 [38]	Yes	>90	5×/week for the time necessary to expend 400 kcal/session during 43.5 weeks of continuous biking/running/walking/exercise on elliptical machine at 70%–80% HR_max_	DEXA	34.8 ± 11.1 (19)	31.7 ±12.2 (19)	46.9 ± 8.0 (19)	47.0 ± 7.7 (19)	74,744	61
Donnelly, 2013 [38]	Yes	>90	5×/week for the time necessary to expend 600 kcal/session during 43.5 weeks of continuous biking/running/walking/exercise on elliptical machine at 70%–80% HR_max_	DEXA	36.4 ± 7.5 (19)	30.5 ± 10.1 (19)	65.0 ± 7.3 (19)	65.4 ± 7.4 (19)	111,703	51
Donnelly, 2013 [38]	Yes	>90	5×/week for the time necessary to expend 400 kcal/session during 43.5 weeks of continuous biking/running/walking/exercise on elliptical machine at 70%–80% HR_max_	DEXA	34.5 ± 11.6 (18)	31.0 ± 11.4 (18)	64.4 ± 9.9 (18)	64.4 ± 9.2 (18)	74,744	56
Dowdy, 1985 [39]	No	≥90	3×/week for 45 min during 10 weeks of continuous aerobic dance at 77% HR_reserve_	HW	19.3 ± 6.4 (18)	19.7 ± 5.8 (18)	43.8 ± 3.1 (18)	44.1 ± 2.3 (18)	11,525	146
Earnest, 2013 [40]	No	N/A	3–4×/week during 12 weeks of continuous running/walking at 50%–70% VO_2max_ and running/walking interval between 90% and 95% VO_2max_ with recuperation at 50% VO_2max_	DEXA	27.5 ± N/A (21)	26.1 ± N/A (21)	66.4 ± N/A (21)	65.5 ± N/A (21)	12,096	−15
Earnest, 2013 [40]	No	N/A	3–4×/week during 12 weeks of continuous running/walking at 50%–70% VO_2max_	DEXA	28.3 ± N/A (16)	27.8 ± N/A (16)	70.6 ± N/A (16)	69.9 ± N/A (16)	12,096	50
Glisezinski, 2003 [41]	Yes	N/A	5×/week for 60 min during 17 weeks of continuous running/biking at a VO_2max_ that increased from 50%–85%	DEXA	20.4 ± N/A (11)	19.0 ± N/A (11)	69.1 ± N/A (11)	68.6 ± N/A (11)	58,785	77
Glowacki, 2004 [42]	Yes	N/A	2–3×/week for 20–40 min during 12 weeks of continuous running at 65%–80% HR_reserve_	HW	19.2 ± N/A (12)	17.3 ± N/A (12)	68.7 ± 9.5 (12)	69.5 ± 9.3 (12)	13,210	−25
Grediagin, 1995 [24]	No	100	4×/week during 12 weeks of continuous exercise on a treadmill at 80% VO_2max_	HW	21.2 ± N/A (6)	18.9 ± N/A (6)	47.0 ± N/A (6)	48.9 ± N/A (6)	14,400	−24
Grediagin, 1995 [24]	No	100	4×/week during 12 weeks of continuous exercise on a treadmill at 50% VO_2max_	HW	21.3 ± N/A (6)	19.0 ± N/A (6)	47.4 ± N/A (6)	48.2 ± N/A (6)	14,400	−39
Hardman, 1992 [43]	No	N/A	≥3×/week for ˃20 min during 52 weeks of continuous brisk walking	HW	23.7 ± 1.5 (28)	24.7 ± 1.6 (28)	40.3 ± N/A (28)	39.6 ± N/A (28)	44,726	125
Hinkleman, 1993 [44]	Yes	N/A	5×/week for 45 min during 15 weeks of continuous walking at 60% HR_reserve_	HW	28.1 ± 1.4 (18)	28.0 ± 1.3 (18)	48.4 ± 0.9 (18)	48.5 ± 0.9 (18)	20,139	96
Juneau, 1987 [45]	No	N/A	5×/week for 47 min during 24 weeks of continuous exercise (N/A) at 50%–66% VO_2max_	HW	17.9 ± N/A (28)	14.0 ± N/A (28)	61.5 ± 8.0 (28)	63.9 ± 13.0 (28)	38,160	15
Juneau, 1987 [45]	No	N/A	5×/week for 54 min during 24 weeks of continuous exercise (N/A) at 50%–66% VO_2max_	HW	17.8 ± N/A (24)	16.6 ± N/A (24)	46.0 ± 5.0 (24)	46.8 ± 4.0 (24)	30,960	68
Kirk, 2003 [46]	Yes	89.6	3–5×/week for 20–45 min during 70 weeks of continuous biking/walking/aerobic exercise in water at a VO_2max_ that increased from 55%–70%	HW	27.4 ± 7.1 (25)	27.2 ± 7.9 (25)	49.5 ± 5.8 (25)	50.4 ± 5.8 (25)	118,837	100
Kirk, 2003 [46]	Yes	90.3	3–5×/week for 20–45 min during 70 weeks of continuous biking/walking/aerobic exercise in water at a VO_2max_ that increased from 55%–70%	HW	26.8 ± 6.8 (16)	21.9 ± 5.5 (16)	67.1 ± 8.3 (16)	66.9 ± 7.8 (16)	177,717	74
Krustrup, 2010 [47]	No	90	2×/week for 60 min during 16 weeks of soccer at 83% HR_max_	DEXA	25.6 ± 1.4 (21)	24.2 ± 1.5 (21)	42.5 ± 1.2 (21)	43.9 ± 1.3 (21)	16,055	33
Krustrup, 2010 [47]	No	92.5	2×/week for 60 min during 16 weeks of continuous running at 82% HR_max_	DEXA	22.0 ± 1.7 (17)	20.9 ± 1.6 (17)	41.6 ± 0.8 (17)	42.9 ± 0.8 (17)	16,055	50
Krustrup, 2009 [48]	No	92	2.3×/week for 60 min during 12 weeks of soccer at 82% HR_max_	DEXA	19.9 ± 2.4 (12)	17.2 ± 2.1 (12)	57.7 ± 2.2 (12)	59.4 ±1.9 (12)	19,783	−13
Krustrup, 2009 [48]	No	100	2.5×/week for 60 min during 12 weeks of continuous running at 82% HR_max_	DEXA	20.7 ± 2.7 (10)	19.0 ± 2.6 (10)	61.3 ± 2.8 (10)	61.9 ± 2.7 (10)	21,503	31
Lee, 2009 [49]	Yes	100	3×/week for 25 min during 6 weeks of continuous running at 60% VO_2max_ and then 4×/week for 40 min during the following 6 weeks of continuous running at 80% VO_2max_	HW	12.1 ± 1.4 (9)	11.2 ± 1.4 (9)	61.7 ± 2.0 (9)	62.1 ± 2.0 (9)	18,615	58
Moro, 2005 [50]	Yes	≥90	5×/week for 45 min (mos1–2) and 60 min (months 3–4) during 17.4 weeks of continuous running/biking at 50%–85% VO_2max_	DEXA	20.2 ± N/A (10)	18.6 ± N/A (10)	70.1 ± N/A (10)	68.7 ± N/A (10)	52,038	68
Mougios, 2006 [25]	Yes	N/A	4×/week during 13 weeks of continuous running at 72% VO_2max_	HW	21.1 ± 2.9 (7)	18.8 ± 2.3 (7)	42.9 ± 4.7 (7)	43.4 ± 4.7 (7)	18,500	−12
Mougios, 2006 [25]	Yes	N/A	4×/week during 13 weeks of continuous running/walking at 45% VO_2max_	HW	23.0 ± 5.7 (7)	20.0 ± 5.9 (7)	45.7 ± 4.2 (7)	45.4 ± 4.6 (7)	18,500	−54
Nishida, 2010 [51]	Yes	100	5×/week for 60 min during 12 weeks of continuous biking at a VO_2max_ that increased from 36.8%–54.8%	HW	9.1 ± N/A (6)	8.9 ± N/A (6)	57.3 ± N/A (6)	57.6 ± N/A (6)	25,304	95
Nordby, 2012 [52]	Not always	85.6	3.5×/week for 51.4 min during 12 weeks of continuous biking at 65% HR_reserve_ and biking/running/rowing/elliptic machine interval at 85% HR_reserve_	DEXA	28.5 ± 1.4 (12)	20.8 ± 1.7 (12)	66.0 ± 2.0 (12)	67.8 ± N/A (12)	24,205	−186
Nybo, 2010 [53]	No	N/A	2.5×/week for 60 min during 12 weeks of continuous running at 65% VO_2max_	DEXA	21.1 ± N/A (9)	19.5 ± N/A (9)	61.3 ± 2.8 (9)	61.9 ± 2.7 (9)	20,454	32
Polak, 2006 [54]	Not always	N/A	5×/week for 45 min during 12 weeks of continuous biking/gymnasium exercise with an increased from 50% to 60% and to 65% VO_2peak_ every 3 weeks	DEXA	34.3 ± N/A (25)	30.2 ± N/A (25)	54.2 ± N/A (25)	53.1 ± N/A (25)	17,965	−120
Rosenkilde, 2012 [9]	No	99	6.2×/week for 30 min during 10 weeks of continuous biking/running at 66% VO_2peak_	DEXA	30.0 ± 4.6 (18)	26.0 ± N/A (18)	63.3 ± 6.9 (18)	63.6 ± N/A (18)	21,105	−76
Rosenkilde, 2012 [9]	No	96	6.2×/week for 55 min during 10 weeks of continuous biking/running at 67% VO_2peak_	DEXA	27.4 ± 4.2 (18)	23.6 ± N/A (18)	64.0 ± 5.7 (18)	65.0 ± N/A (18)	41,139	17
Ruby, 1996 [55]	Yes	≥95	4×/week for 45 min during 10 weeks of continuous running at 70%–80% HR_reserve_	HW	12.0 ± N/A (6)	10.5 ± N/A (6)	46.2 ± N/A (6)	46.5 ± N/A (6)	16,686	21
Ruby, 1996 [55]	Yes	≥95	4×/week for 45 min during 10 weeks of continuous biking at 70%–80% HR_reserve_	HW	14.5 ± N/A (6)	13.5 ± N/A (6)	47.1 ± N/A (6)	47.7 ± N/A (6)	14,936	47
Ruby, 1996 [55]	Yes	≥95	4×/week for 45 min during 10 weeks of continuous biking/running at 70%–80% HR_reserve_	HW	17.5 ± N/A (6)	17.4 ± N/A (6)	44.9 ± N/A (6)	45.4 ± N/A (6)	16,330	101
Santiago, 1995 [56]	Yes	91	4×/week during 38 weeks of continuous walking at 72% HR_max_	HW	18.4 ± N/A (16)	17.0 ± N/A (16)	46.0 ± N/A (16)	46.4 ± N/A (16)	52,440	76
Sedlock, 2010 [57]	Yes	100	3–4×/week for 25–40 min during 12 weeks of continuous running at a VO_2max_ that increased from 60%–80%	HW	12.1 ± 1.4 (9)	11.2 ± 1.4 (9)	61.7 ± 2.0 (9)	62.1 ± 2.0 (9)	16,905	54
Sijie, 2012 [58]	Yes	N/A	5×/week for 27 min during 12 weeks of walking (12 min)/running (15 min) interval at 50% and 85% VO_2peak_	DEXA	29.9 ± N/A (17)	24.7 ± N/A (17)	43.8 ± N/A (17)	42.8 ± N/A (17)	14,385	−248
Sijie 2012 [58]	Yes	N/A	5×/week for 40 min during 12 weeks of continuous running/walking at 50% VO_2peak_	DEXA	30.5 ± N/A (16)	27.2 ± N/A (16)	43.7 ± N/A (16)	42.6 ± N/A (16)	15,003	−114
Snyder 1997 [59]	Not always	82.6	5×/week for 3 × 10 min during 32 weeks of continuous walking at 52% HR_reserve_	HW	36.7 ± 14.5 (13)	37.2 ± 14.7 (13)	50.6 ± 9.8 (13)	49.9 ± 10.1 (13)	19,554	128
Suter, 1995 [60]	No	N/A ^1^	4×/week for 30 min during 26 weeks of continuous running at 75% VO_2max_	DEXA	16.6 ± 6.1 (12)	15.7 ± 6.4 (12)	52.9 ± 6.6 (12)	53.5 ± 6.3 (12)	36,433	80
Tan, 2012 [61]	Yes	≥88	5×/week for 40 min during 8 weeks of continuous running at 54% VO_2max_	DEXA	31.0 ± 4.6 (29)	27.0 ± 4.0 (29)	39.5 ± 4.9 (29)	39.4 ± 4.4 (29)	10,797	−250
Trapp, 2008 [62]	No	100	3×/week for 20 min during 15 weeks of biking interval at 53.2% VO_2peak power output_	DEXA	22.2 ± 30.0 (11)	19.7 ± 2.6 (11)	41.1 ± N/A (11)	42.1 ± N/A (11)	9915	−119
Trapp, 2008 [62]	No	100	3×/week for 30 min during 15 weeks of continuous biking at 60% VO_2peak_	DEXA	18.4 ± 2.2 (8)	18.8 ± 2.1 (8)	41.4 ± N/A (8)	40.9 ± N/A (8)	8,673	150
Van Aggel-Leijssen, 2002 [63]	Yes	89.4	3×/week for 57 min during 12 weeks of continuous biking at 40% VO_2max_	HW	32.7 ± N/A (8)	32.4 ± N/A (8)	70.0 ± 9.6 (8)	70.7 ± 8.7 (8)	12,600	87
Van Aggel-Leijssen, 2002 [63]	Yes	92.6	3×/week for 33 min during 12 weeks of continuous biking at 70% VO_2max_	HW	32.7 ± N/A (8)	33.3 ± N/A (8)	72.8 ± 5.4 (8)	71.8 ± 6.7 (8)	13,104	148
Van Aggel-Leijssen, 2001 [64]	Yes	81	3×/week for 57 min during 12 weeks of continuous biking at 40% VO_2max_	HW	41.2 ± N/A (8)	41.7 ± N/A (8)	50.0 ± 2.4 (8)	49.5 ± 2.7 (8)	9000	162
Van Aggel-Leijssen, 2001 [64]	Yes	88	3×/week for 57 min during 12 weeks of continuous biking at 40% VO_2max_	HW	37.1 ± N/A (7)	37.5 ± N/A (7)	49.4 ± 3.7 (7)	49.6 ± 3.8 (7)	8892	158
Wilmore, 1980 [65]	No	99.1	3×/week for 30 min during 20 weeks of continuous biking at 75% HR_reserve_	HW	19.3 ± N/A (9)	18. 0 ± N/A (9)	66.4 ± N/A (9)	67.4 ± N/A (9)	23,978	56
Wilmore, 1980 [65]	No	93.3	3×/week for 30 min during 20 weeks of continuous running at 75% HR_reserve_	HW	16.2 ± N/A (9)	14.5 ± N/A (9)	63.6 ± N/A (9)	63.5 ± N/A (9)	24,239	34

The values are the mean ± SD. The number in parentheses represents the number of participants tested. Notes: HRR_max_, heart rate reserve maximal; HR_max_, heart rate maximal; HW, hydrostatic weighing; DEXA, dual X-ray absorptiometry; BP, Bod Pod; ^1^ compliance minimum of 60 min/week.

**Figure 2:**
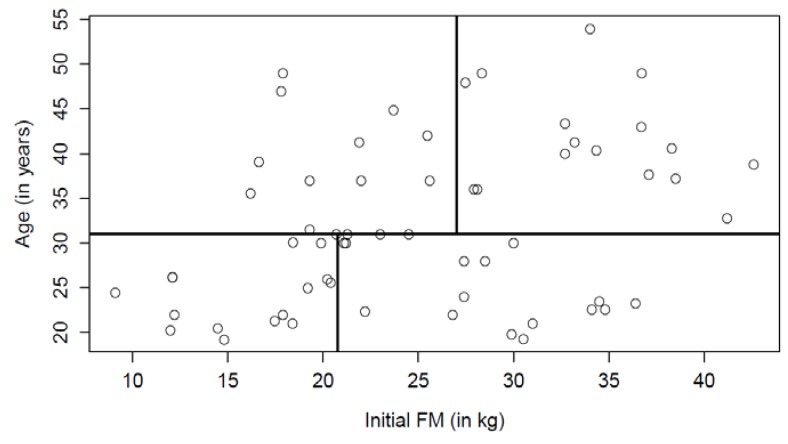
Correlation between age and initial FM. The median of age = 31 years old; the median for initial FM in studies involving younger subjects = 20.8 kg; and the median for older subjects = 27.5 kg. There were *n* = 15 studies in all groups, except for the group of older subjects with a high initial FM (*n* = 16 studies).

The multiple linear regression model suggested that 48% of the variance of the degree of energy compensation is explained by the interaction between initial FM, the age of individuals and according to studies of different intervention duration (*p* < 0.0001). To describe the interactions, initial FM and age were dichotomized. Results suggested that studies involving older subjects presented larger initial FM on average compared to studies that involved younger subjects (Figure 2). To account for this relationship, studies were partitioned according to the median age (31 years old). Then, the medians for initial FM in studies involving younger subjects (20.8 kg) and older subjects (27.5 kg) were found. There were *n* = 15 studies in all groups, except for the group with older subjects with a high initial FM (*n* = 16 studies).

**Figure 3:**
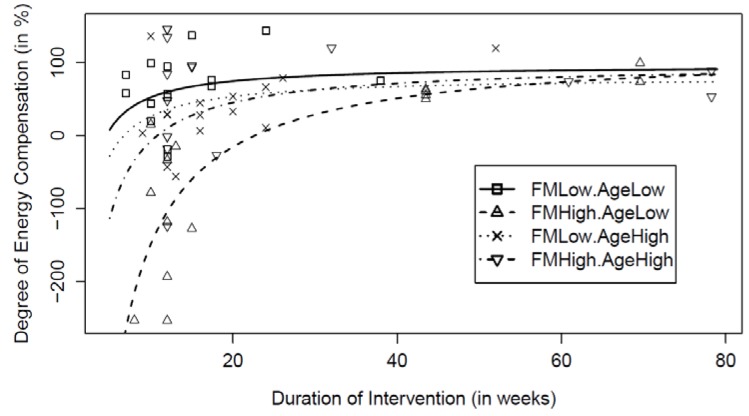
Degree of energy compensation illustrated as the interaction between age and initial FM of individuals, as well as with the duration of each exercise intervention. Each exercise intervention study is represented by a symbol.

Figure 3 illustrates these interactions. Overall, the degree of energy compensation is highly variable for interventions of shorter duration, while it is near 84% for interventions of longer duration (about 80 weeks). At 10 weeks, significant differences were noticed between younger with lower FM and older individuals with higher FM (*p* < 0.0001). Furthermore, significant differences were noticed for younger individuals with lower FM and higher FM (*p* < 0.0001), as well as between younger individuals with higher FM and older individuals with lower FM (*p* < 0.0001). For younger individuals with smaller initial FM, it is shown that the degree of energy compensation is maintained at about 97% independently of the intervention duration. The degree of energy compensation is also similar for varying durations of the exercise interventions for older individuals with smaller initial FM (degree of energy compensation = 81%). For younger and older individuals with higher FM, the equations were respectively:

*Energy compensation* (%) = 117.5 − 2663.6/ *Duration* (3)

*Energy compensation* (%) = 97.8 − 1055.2/ *Duration* (4)

## 4. Discussion

This systematic review aimed to determine the energy compensation following aerobic exercise interventions that did not include dietary modifications as part of the interventions. More specifically, the independent predictors of energy compensation and their interactions were investigated. Energy compensation in all included studies was determined using ExEE and body composition changes. The resulting mean energy compensation for these studies was 18%. Forty-eight percent of the variance in energy compensation was explained by the interaction between initial FM, age and intervention duration. Our analyses also revealed that negative energy compensation induced through exercise seems to be present for short-term interventions, but tends to subside when exercise interventions are prolonged. 

For exercise interventions of shorter duration (less than 25 weeks), the results of the analyses suggested that a greater weight loss was achieved in younger individuals with higher initial FM. In contrast, this review highlighted that younger individuals with lower initial FM presented a positive level of energy compensation. Even if the effects of adiposity [14,15,16,17] and age [18,19,20] on energy compensation have been separately investigated, the findings of the possible association between initial FM, age and duration of the exercise interventions on energy compensation is a novel contribution of this paper and warrants further investigation.

The explanation as to why longer exercise interventions would lead to higher energy compensation is intriguing. It could be speculated that the energy compensation is explained by an increase in fatigue or a decrease in non-structured physical activity (NSPA) over time [20,66,67,68,69]. Conversely, as fitness increases, it could also be speculated that the same exercise would be less tiring. Moreover, it is also possible that a longer exercise intervention would increase hunger, which would ultimately lead to higher EI [70,71,72]. However, since we did not have access to EI or NSPA (*i.e.*, EI and NSPA were not available over the 61 groups included in the final analysis), it is impossible to determine to what extent these factors contributed to these observations. Therefore, the specific role of the modifications of EI and EE in response to long-term exercise interventions likely needs to be more closely inspected to fully capture their respective contribution to energy compensation.

Our analyses show that sex did not contribute to the variance in energy compensation. It has been suggested that following exercise, energy compensation would be greater in women [11]. However, the explanation came from the fact that energy expenditure from exercise was lower in women when compared to men [11,73]. The results from this systematic review are rather in line with the results reported by Caudwell *et al.* [12], McTiernan *et al.* [13] and Donnelly, 2013 [38], who have shown that exercise-induced weight loss is similar between men and women as long as ExEE is equivalent between groups. 

Additionally, the results of this systematic review show that energy compensation does not vary as a function of the frequency, dose and/or the intensity of ExEE. This conclusion is discordant with the results from the Studies of a Targeted Risk Reduction Intervention through Defined Exercise (STRRIDE) [74]. In this study, overweight men and women running 32 km/week at 65%–80% of their VO_2max_ lost significantly more weight and fat mass when compared to the ones who ran 19.2 km/week at 65%–80% or 40%–50% of their VO_2max_. One of the major finding from that study suggests the existence of a dose response to exercise. Nevertheless, in overweight young men expending 300 or 600 kcal per day, the results suggest the same level of weight loss, which emphasised a proportional increase in energy compensation with the dose of exercise [9]. Similarly, the Dose Response to Exercise in Women aged 45–75 years (DREW) study proposed a lower than predicted weight loss in overweight women expending 12 kcal/kg/week when compared to those expending 4 or 8 kcal/kg/week [23]. Furthermore, some studies have shown that body weight decreased significantly following a lower intensity (LI) exercise intervention compared to a higher intensity (HI) exercise intervention [24,25], while others have found no difference between high- and low-intensity exercise interventions [44,63,64]. Finally, as concluded by Thomas *et al.* [6] in their systematic review showing a “small magnitude of weight loss” in response to ExEE, it is not impossible that the small amount of weight loss following exercise interventions could be caused by the small dose of ExEE. 

This systematic review is limited to an adult population and cannot be extended to youth or elderly individuals. The different methods used to measure body composition (*i.e.*, DEXA, hydrostatic weighing and bod pod) could have influenced the results due to their varying degree of accuracy. In addition, the possibility that some participants included in the different studies might have followed a diet throughout the interventions cannot be excluded even if studies that included a formal dietary intervention were excluded from the analyses. The dichotomisation of the variable intensity could have reduced the power of the statistical analyses. However, only considering the studies that reported the intensity of the exercise based on VO_2peak_ would have reduced the number of groups included from 101 to 54. It is also important to consider that only a few studies of longer duration were included in this analysis. Other potential limitations of this systematic review are that individuals included in the different studies were not all sedentary and not all papers mentioned a stable body weight as an inclusion criterion. Some studies also reported a high dropout rate (N = 6/101). Since one of the factors for dropping out of such interventions is modest early weight losses [75], these individuals could have potentially inflated the compensation to ExEE if they had continued the exercise intervention program. ExEE was either provided in the articles or was calculated from available data. Even in the cases where ExEE was provided in the studies, it is important to note that it was not directly measured throughout all exercise sessions. In those studies where we had to calculate ExEE, it was assumed that for each exercise session, the energy cost was 5 kcal/LO_2_, and we employed the best available information to provide the most accurate calculation of ExEE. In either case, ExEE was not measured at every training session for the studies included in this review, so it could obviously be over- or under-estimated. As such, the fact remains that the exercise compensation results presented herein stem from an estimation of ExEE, and the findings need to be interpreted accordingly. Furthermore, the compendium of physical activities (2011) was used to estimate the ExEE when needed, which could have under-/over-estimated the ExEE in some cases. Excess post-exercise oxygen consumption, even if not included in the analyses, would inflate energy expenditure, thus further increasing the energy compensation phenomenon already observed from our findings. As for the training, not all sessions were performed under supervision, and the compliance for most studies was not reported. For example, it is possible to speculate that not all exercise sessions lasted the same amount of time or at the stated intensity throughout the intervention, reducing the total amount of ExEE and, thus, inflating the energy compensation. 

## 5. Conclusions

In conclusion, results from this systematic review show that initial FM, age and the duration of the intervention are the most significant predictors of energy compensation. The current findings demonstrate that when negative energy compensation is achieved with ExEE, it can only be maintained over a relatively short time span. In contrast, longer term exercise interventions are accompanied by levels of energy compensation that hover around 84%, which could be related to the more potent expression of compensatory mechanisms that oppose the decrease of body energy stores over longer periods of time. In order to fully comprehend exercise-induced energy compensation, future studies should include accurate determinations of EI and EE in the study designs.

## Appendix

### Appendix 1. MEDLINE search strategy.

1 exercise/or running/or jogging/or swimming/or walking/
2 Motor activity/
3 Physical Fitness/
4 Exercise Therapy/
5 exp Sports/
6 Dancing/
7 exercis*.tw.
8 physical activit*.tw.
9 vigorous activit*.tw.
10 physical training.tw.
11 exertion.tw.
12 (aerobic* or walking or jogging or swimming or cycling or bicycling or running).tw.
13 (fitness adj3 (class* or regime* or program*)).tw.
14 danc*.tw.
15 endurance training.tw.
16 or/1–15
17 Energy Metabolism/
18 (energy adj3 spent).tw.
19 (energy adj3 output).tw.
20 (energy adj2 expend*).tw.
21 (calori* adj3 burn*).tw.
22 (calori* adj3 expend*).tw.
23 Oxygen Consumption/
24 (oxygen adj3 consum*).tw.
25 (O2 adj3 consum*).tw.
26 “(VO(2 max))”.tw.
27 VO2max.tw.
28 (VO2 adj2 peak).tw.
29 (oxygen adj2 uptake).tw.
30 (oxygen adj2 intake).tw.
31 (aerobic capacity adj3 max*).tw.
32 or/17–31
33 body composition.mp.
34 Body Fat Distribution/
35 (fat adj3 mass).tw.
36 body fat.tw.
37 (fat adj3 percentage).tw.
38 body weight/
39 body weight changes/
40 weight gain/ or weight loss/
41 obesity/ or overweight/
42 normal weight.tw.
43 lean body.tw.
44 or/33–43
45 randomized controlled trial.pt.
46 controlled clinical trial.pt.
47 (randomized or randomly).tw.
48 trial.ti.
49 (control* adj3 (study or studies or trial)).tw,hw.
50 time series.tw.
51 (pre test or pretest or posttest or post test).tw.
52 quantitative.tw.
53 cohort studies/
54 or/45–53
55 exp animals/ not humans.sh.
56 54 not 55
57 16 and 32 and 44
58 56 and 57
59 limit 58 to (English or French)
